# Crystal Structures of Two Aminoglycoside Kinases Bound with a Eukaryotic Protein Kinase Inhibitor

**DOI:** 10.1371/journal.pone.0019589

**Published:** 2011-05-09

**Authors:** Desiree H. Fong, Bing Xiong, Jiyoung Hwang, Albert M. Berghuis

**Affiliations:** 1 Department of Biochemistry, McGill University, Montreal, Quebec, Canada; 2 Groupe de Recherche GRASP, McGill University, Montreal, Quebec, Canada; 3 Department of Microbiology and Immunology, McGill University, Montreal, Quebec, Canada; National Institute for Medical Research, Medical Research Council, London, United Kingdom

## Abstract

Antibiotic resistance is recognized as a growing healthcare problem. To address this issue, one strategy is to thwart the causal mechanism using an adjuvant in partner with the antibiotic. Aminoglycosides are a class of clinically important antibiotics used for the treatment of serious infections. Their usefulness has been compromised predominantly due to drug inactivation by aminoglycoside-modifying enzymes, such as aminoglycoside phosphotransferases or kinases. These kinases are structurally homologous to eukaryotic Ser/Thr and Tyr protein kinases and it has been shown that some can be inhibited by select protein kinase inhibitors. The aminoglycoside kinase, APH(3′)-IIIa, can be inhibited by CKI-7, an ATP-competitive inhibitor for the casein kinase 1. We have determined that CKI-7 is also a moderate inhibitor for the atypical APH(9)-Ia. Here we present the crystal structures of CKI-7-bound APH(3′)-IIIa and APH(9)-Ia, the first structures of a eukaryotic protein kinase inhibitor in complex with bacterial kinases. CKI-7 binds to the nucleotide-binding pocket of the enzymes and its binding alters the conformation of the nucleotide-binding loop, the segment homologous to the glycine-rich loop in eurkaryotic protein kinases. Comparison of these structures with the CKI-7-bound casein kinase 1 reveals features in the binding pockets that are distinct in the bacterial kinases and could be exploited for the design of a bacterial kinase specific inhibitor. Our results provide evidence that an inhibitor for a subset of APHs can be developed in order to curtail resistance to aminoglycosides.

## Introduction

The waning prospect of an effective treatment for bacterial infections due to the emergence and spread of resistance to antibiotics in pathogens has been exacerbated by the lack of novel antibacterials being introduced to the market [1]. An alternative and parallel approach in supporting the mitigation of the antibiotic resistance problem is to develop adjuvants that could interfere with the mechanism of resistance and hence restore the action of antibiotics [2]. Such a strategy has been effectively employed to combat resistance to β-lactams due to β-lactamase activity [3]. For aminoglycosides, a group of antibiotics used to treat serious nosocomial infections, the main mechanism of resistance is via the enzymatic inactivation of the drug by acetyltransferases, nucleotidyltransferases, or phosphotransferases [4]. This implies that inhibitors of these enzymes could be exploited for the development of drug-adjuvant therapy [5], [6]. Among the three types of aminoglycoside-modifying enzymes, aminoglycoside phosphotransferases or kinases (APHs) yield the highest levels of resistance thereby providing a rationale for focusing inhibitor development for these specific resistance factors [7].

**Figure 1:**
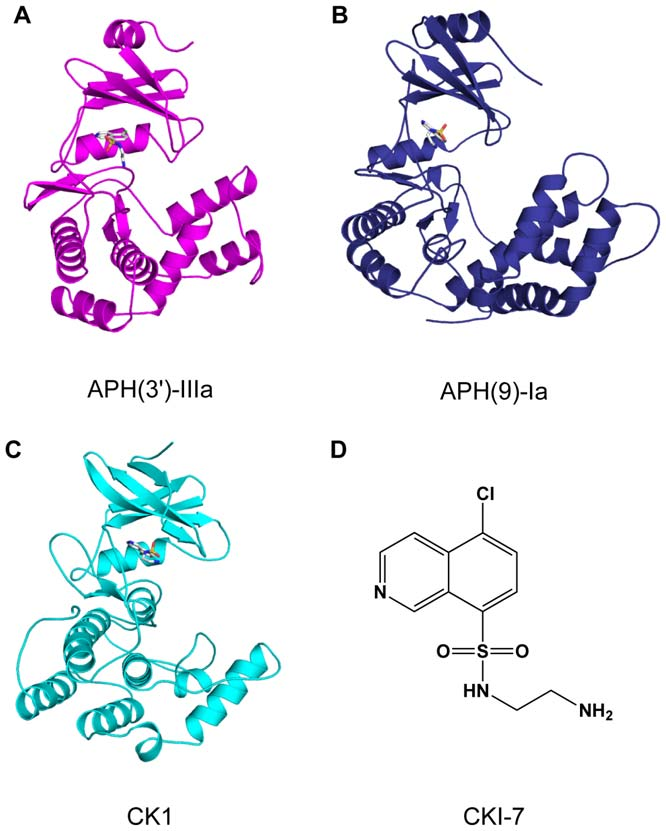
Crystal structures of CKI-7-bound kinases. (A) APH(3′)-IIIa, (B) APH(9)-Ia, and (C) CK1 (PDB 2CSN). The enzymes are shown in cartoon representation and the inhibitors are drawn as sticks. (D) Chemical structure of CKI-7.

The investigation of APH inhibitors that target the ATP-binding pocket was facilitated by the structural similarities between the aminoglycoside resistance enzyme APH(3′)-IIIa and serine/threonine and tyrosine eukaryotic protein kinases (ePKs), especially in the N-terminal lobe [8] (Figure 1A,C). It was subsequently shown that APH(3′)-IIIa can be inhibited by protein kinase inhibitors of the isoquinolinesulfonamide family and they are competitive with ATP-binding [9]. For example, the protein kinase inhibitor *N*-(2-aminoethyl)-5-chloro-isoquinoline-8-sulfonamide (CKI-7) (Figure 1D) has an inhibition constant of 65 µM for APH(3′)-IIIa. Unfortunately, these compounds are only able to inhibit the resistance enzymes *in vitro* and cannot rescue the function of aminoglycosides in enterococcal strains harboring the *aph(3′)-IIIa* gene [9]. Nonetheless, this study identified lead compounds for adjuvant development aimed at reversing APH mediated resistance to aminoglycosides.

X-ray structures of several members in the APH family have since been determined [8], [10], [11], [12], [13], [14]. However, APH(3′)-IIIa remains the most extensively studied due to its broad substrate spectrum [9], [15], [16], [17], [18], [19]. The crystal structure of APH(3′)-IIIa in the apo, ADP- or AMP-PNP-bound forms [8], [20], as well as its ternary complex of three structurally dissimilar aminoglycosides [10], [21] are known. Perhaps the most different among the APHs examined structurally is APH(9)-Ia (e.g. 9% sequence identity with APH(3′)-IIIa). APH(9)-Ia is an atypical APH which phosphorylates only one aminoglycoside, spectinomycin, that is distinct from the other aminoglycoside antibiotics. Its apo, AMP-bound and the ternary structures have been determined, making it the second structurally most studied member of the APH family [11]. Combined, these studies reveal that although members of the APH family share low similarities in sequence and their ligand specificity varies greatly, their overall three-dimensional fold is homologous to each other and to that of ePKs (Figure 1A–C).

To further advance the development of APH inhibitors, we describe here the three-dimensional structure of the APH(3′)-IIIa and APH(9)-Ia in complex with CKI-7 (PDB accession codes 3Q2J and 3Q2M, respectively). These inhibitor bound crystal structures of APHs represent the first structures of a eukaryotic protein kinase inhibitor complexed to enzymes that are not eukaryotic protein kinases. Comparison of the inhibitor-bound APH(3′)-IIIa and APH(9)-Ia complexes with the nucleotide-bound APH(3′)-IIIa and APH(9)-Ia, as well as the CKI-7-bound casein kinase 1 (CK1) reveals the different inhibitor binding modes as well as topological features that could be exploited in the development of inhibitors with enhanced affinity and selectivity for APH enzymes.

## Results and Discussion

### Inhibition of APHs by CKI-7

Previously, details on the inhibition of APH(3′)-IIIa by CKI-7 have been reported (K_i_ = 66.1±7.5 µM) [9]. Here we show that the atypical APH, APH(9)-Ia, is also affected by this protein kinase inhibitor. Paralleling the APH(3′)-IIIa result, CKI-7 was found to inhibit APH(9)-Ia (K_i_ = 159±11 µM) in a competitive fashion with respect to ATP, although 2.5 times less effectively. These results suggest that the CKI-7 scaffold may be exploited for the development of broad-spectrum APH inhibitors. This possibility is further reinforced by the observation that a third APH enzyme, APH(2″)-Ia is similarly inhibited by this compound (K_i_ = 87.1±17.8 µM) [9]. However, the inhibition constants for CKI-7 are high, especially when compared to that obtained for the ePK CK1, which is an order of magnitude lower (K_i_ = 8.5 µM) [22]. Nonetheless, at present CKI-7 is the only compound identified that is able to inhibit the ATP-binding site of several APH enzymes.

### Crystal structures of APH•CKI-7 complexes

**Figure 2:**
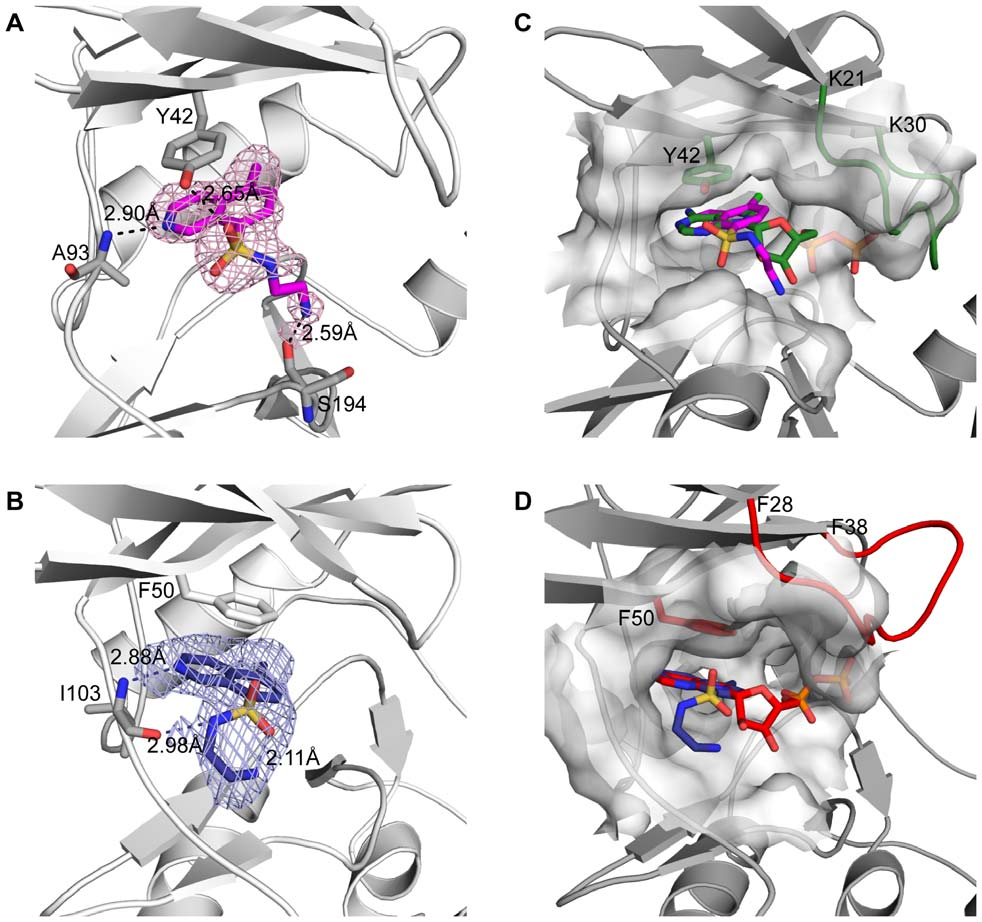
Nucleotide/inhibitor binding sites of APH(3′)-IIIa and APH(9)-Ia. The CKI-7- and nucleotide-bound APH(3′)-IIIa are shown in cartoon representation in panels A and C respectively; The CKI-7- and nucleotide-bound APH(9)-Ia are shown in cartoon representation in panels B and D, respectively. (A) CKI-7 bound to APH(3′)-IIIa in magenta sticks and its simulated annealing F_o_-F_c_ omit map, contoured at 2.5σ, are shown. Tyr42, which forms stacking interactions with the isoquinoline, and Ala93, which hydrogen bonds with the inhibitor, are shown in sticks. (B) CKI-7 bound to APH(9)-Ia in dark blue sticks and its simulated annealing F_o_-F_c_ omit map, contoured at 2.5σ, are shown. Phe50 whose aromatic ring stacks with the isoquinoline, and Ile103, which forms hydrogen bond interactions with the inhibitor, are shown in sticks. (C,D) Comparison of the inhibitor- and nucleotide-bound APHs. The homologous ePK gly-rich loop is highlighted in green for nucleotide-bound APH(3′)-IIIa (C) and red for APH(9)-Ia (D). Tyr42 in APH(3′)-IIIa and Phe50 in APH(9)-Ia, that stacks the adenine ring of the nucleotide are shown in sticks and their carbon atoms are colored green for APH(3′)-IIIa (C) and red for APH(9)-Ia (D). The CKI-7-binding sites are delineated by surface representation in their respective enzymes. CKI-7 bound to APH(3′)-IIIa and APH(9)-Ia are colored as in panels A and B. CKI-7 binds to the same region and in an analogous manner as the nucleotide to each of the enzyme. The phosphates of the nucleotide are buried in the surface representing the homologous gly-rich loop in the presence of CKI-7.

To further examine the suitability of the CKI-7 scaffold for inhibitor development, we determined the crystal structures of APH(3′)-IIIa and APH(9)-Ia in complex with CKI-7 (Figure 1A,B). The APH(3′)-IIIa-CKI-7 inhibitor complex was crystallized in the space group P2_1_2_1_2_1_ with two inhibitor-bound enzyme molecules in the asymmetric unit, analogous to the nucleotide bound enzyme complexes [8], [20]. The structure has been refined to 2.15 Å with an R_cryst_ of 0.189 and R_free_ of 0.235. The CKI-7-bound APH(9)-Ia complex was crystallized in space group P3_1_21 with one molecule per asymmetric unit. This structure has been refined to 2.9 Å and a final R_cryst_ and R_free_ of 0.222 and 0.279, respectively. Comparison of these crystal structures with the apo, nucleotide-bound, and aminoglycoside and nucleotide-bound states, available for both APH(3′)-IIIa and APH(9)-Ia, reveals that the CKI-7-bound enzyme conformations most closely resemble that of the binary nucleotide-bound states. This is to be expected, as CKI-7 is competitive with ATP. However, some differences can also be noted between the APH-CKI-7 bound states and that of the APH-nucleotide bound states, most notably in the region designated as the glycine-rich loop in ePKs. In APH(3′)-IIIa the homolog of the glycine-rich loop are residues 21-27 (Figure 2C). In the nucleotide-bound enzyme structures, the loop is positioned above the phosphate moieties of the nucleotide [20], whereas, in the APH(3′)-IIIa-CKI-7 structure, the tip of the loop points back into the phosphate-binding area, delimiting the nucleotide-binding pocket, reminiscent of what is seen in the apo state of APH(3′)-IIIa [20]. For APH(9)-Ia, this same loop (residues 28–34) also adopts a different conformation in the APH(9)-Ia-CKI-7 structure compared to the nucleotide-bound state (Figure 2D). In fact, the conformation observed is comparable to what is observed for APH(3′)-IIIa, i.e. the tip of loop in the CKI-7-bound APH(9)-Ia also dips into the phosphate-binding area of the nucleotide binding pocket.

### Inhibitor Binding Site

**Figure 3:**
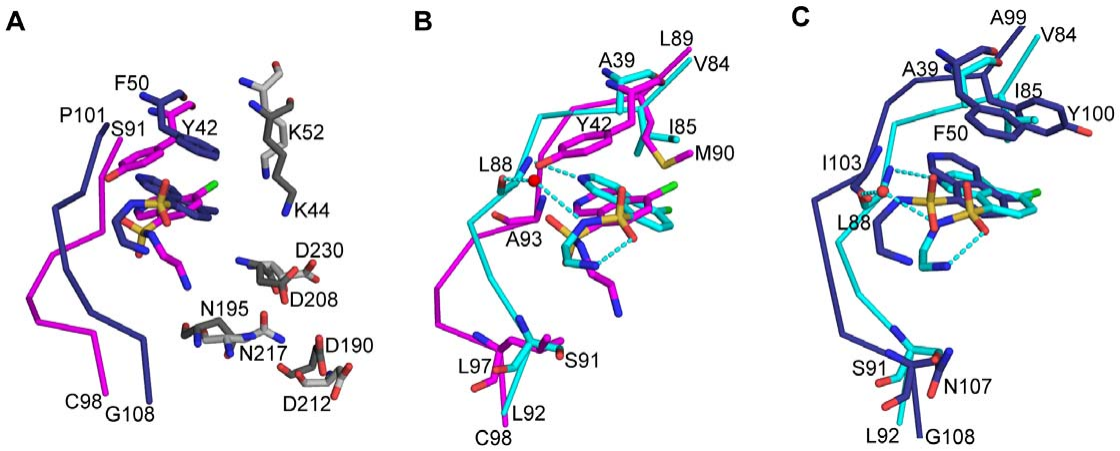
Comparisons of CKI-7 binding to APH(3′)-IIIa, APH(9)-Ia and CK1. The structures are superposed using the conserved residues among the enzymes. (A) Superposition of CKI-7-bound APH(3′)-IIIa and APH(9)-Ia. The conserved active site residues between APH(3′)-IIIa and APH(9)-Ia are colored dark grey and light grey respectively. The APH(3′)-IIIa-bound CKI-7 is shown as magenta sticks and the APH(9)-Ia-bound CKI-7 is shown as dark blue sticks. The rotation between the planes of the isoquinoline rings of CKI-7 bound to APH(3′)-IIIa and APH(9)-Ia is approximately 40°. (B) Superposition of CKI-7-bound APH(3′)-IIIa and CK1. The same degree of rotation as that observed between the isoquinolines of the APH(3′)-IIIa- and APH(9)-Ia-bound CKI-7 is observed here. (C) Superposition of CKI-7-bound APH(9)-Ia and CK1. The CKI-7 isoquinoline rings are coplanar. In panels (B) and (C), APH(3′)-IIIa and APH(9)-Ia are colored as in panel A, and CK1 is colored in cyan. The residues that interact with CKI-7 and those with dissimilar properties from ePK nucleotide-binding site are shown in sticks. Hydrogen bond interactions between CKI-7 and CK1 are illustrated as dash lines. Water molecule is represented by a red sphere.

As expected, for both APH(3′)-IIIa and APH(9)-Ia, the ATP-competitive inhibitor CKI-7 occupies the nucleotide-binding pocket, between the N- and C-terminal lobes. The binding of the inhibitor did not alter the main or side chain conformation of any residues lining the binding pocket in either enzyme, except for the APH homolog of the glycine-rich loop mentioned above. The isoquinoline ring of the inhibitor is buried in the hydrophobic adenine-binding cleft (Figure 2A,B) and its position and orientation mimics that of the adenine ring of the nucleotide (Figure 2C,D). A principal contact between the isoquinoline or adenine ring with APH is the stacking interactions conferred by the aromatic ring side chain of Tyr42 in APH(3′)-IIIa and Phe50 in APH(9)-Ia (Figure 2). The residue at this location is conserved as either a tyrosine or phenylalanine among all APH(3′), APH(9), as well as APH(3″) enzymes, and shown to be important for binding and catalysis [20]. Intriguingly, despite the conserved nature of this residue, the aromatic side chain of Tyr42 of APH(3′)-IIIa points toward the linker region of the protein whereas the side chain of Phe50 in APH(9)-Ia points in the opposite direction toward the ribose- and phosphate-binding pockets (Figure 3A). Accordingly, the adenine ring of the bound nucleotides adopt distinct orientations, differing by a rotation of approximately 40° [11]. The corresponding 40° difference is also observed between the isoquinoline rings of CKI-7 bound to APH(3′)-IIIa or APH(9)-Ia (Figure 3A).

The linker region between the N- and C-terminal lobes of the APH enzymes plays an important role in the binding of the isoquinoline or adenine ring. The N1 and N6 of the adenine ring form hydrogen bond interactions with the main chain amide of Ala93 (APH(3′)-IIIa numbering) and carbonyl of Ser91, respectively [8]. Although the sole cyclic nitrogen in the isoquinoline, N2, and the adenine, N1, are located in different positions of the ring structure, CKI-7 is positioned in such a way that N2 of the isoquinoline overlays with N1 of the adenine ring in the nucleotide. Consequently, an interaction analogous to that between N1 of the adenine and the linker amide is observed between N2 of the isoquinoline and the amide of Ala93 in APH(3′)-IIIa or Ile103 in APH(9)-Ia. The conformation of the linker region between the N- and C-terminal lobes appears to have an impact on the binding orientation of the adenine or isoquinoline ring. Comparison of the linker in the nucleotide- and CKI-7-bound complexes of APH reveals that this segment in APH(3′)-IIIa is located closer to the loop connecting helix α2 and strand β4 behind the adenine-binding pocket, thereby creating a more open nucleotide-binding site. The difference in the positioning of the shorter linker region in APH(9)-Ia renders a deviation in the location of Pro101 and Ile103 from their counterparts in APH(3′)-IIIa, thus leading to a corresponding and necessary change in the binding orientation of the adenine or isoquinoline ring in order to maintain the hydrogen bond between the cyclic nitrogen and the main chain amide.

The remainder of the CKI-7 inhibitor, the aminoethyl-sulfonamide, adopts different conformations when bound to the two APH enzymes. In APH(3′)-IIIa, the aminoethyl-amide adopts an extended conformation and it is situated just beyond the ribose-binding area, toward the solvent exposed opening of the ATP-binding pocket (Figure 2A and 3A). Alternatively, using the terminology of the different compartments in the ATP-binding site of ePK, the aminoethyl-sulfonamide lies adjacent to the ribose-binding pocket, bordering the specificity surface [23] or the entrance pocket [24]. This portion of the inhibitor is more flexible than the isoquinoline ring as reflected by the relatively higher thermal factors. Two hydrogen bonds are observed between this section of the CKI-7 and the APH(3′)-IIIa (Figure 2A). One of which is found between one of the oxygen atoms of the sulfonyl group (O2S) and the hydroxyl group of Tyr42. The second hydrogen bond is formed between the terminal nitrogen of the aminoethyl tail, N2′, and the main chain carbonyl of Ser194. An analogous interaction is observed in the APH(3′)-IIIa-nucleotide complex between the carbonyl of Ser194 and the O3′ of the ribose, which approximately overlaps the N2′ of CKI-7. In APH(9)-Ia, the aminoethyl group of the inhibitor is positioned in the entrance pocket [24] or the solvent-exposed side of the adenine-binding pocket and it points back at itself forming an intramolecular interaction with the equatorial sulfonyl oxygen atom (O2S) (Figure 2B,3A). The inhibitor also makes a second interaction with the linker region of APH(9)-Ia via the N_β_ of the aminoethyl tail and the main chain carbonyl of Ile103. No interactions, direct or water-mediated, are observed between the linker of APH(3′)-IIIa and the aminoethyl of CKI-7 since the linker is one residue longer in APH(3′)-IIIa and is situated over 7 Å away from the binding pocket compared to the equivalent in APH(9)-Ia (Figure 3A).

### Comparison of CKI-7-bound APHs and casein kinase 1

The crystal structure CK1 in complex with CKI-7 has been determined [25] (Figure 1C). Here, CKI-7 also occupies the ATP-binding cleft of CK1, and the overall structures of the inhibitor- and nucleotide-bound CK1 are the same, differing slightly in the glycine-rich loop, analogous to what is observed for APH(3′)-IIIa and APH(9)-Ia. The isoquinoline ring of the inhibitor is coplanar with the adenine moiety of ATP and the aminoethyl-sulfonamide points away from the ribose toward the solvent accessible opening of the binding pocket. When the CKI-7-bound structures of APH(3′)-IIIa, APH(9)-Ia, and CK1 are superposed using the coordinates of conserved active site residues, it is apparent that the plane of the isoquinoline ring in the CK1 structure differs from that observed in APH(3′)-IIIa by a rotation of approximately 40° (Figure 3B) but is nearly coplanar to that in APH(9)-Ia (Figure 3C), analogous to the binding of the adenine ring in the nucleotide-bound enzymes [20]. Furthermore, the linker region of CK1, in particular the N-terminal section which form hydrogen bond contacts with the ring structure of the ligand, superpose well with that of APH(9)-Ia.

The hydrogen bond between the cyclic nitrogen and a main chain amide (Leu88 in CK1) in the linker of the enzyme is also present in the CKI-7-bound CK1 (Figure 3B,C). The equivalent hydrogen bond observed between N1 of adenine and the tethering segment in the three enzymes is conserved in all adenine-binding to ePKs [26]. This hydrogen bond is not unique to isoquinolinesulfonamide type inhibitors binding to the three enzymes discussed here. A majority of ePK crystal structures complexed with an ATP-competitive inhibitor form at least one hydrogen bond with residues in the linker region, mimicking the ones between N1 and/or the exocyclic N6 of the adenine and the enzyme [23]. The significance of the hydrogen bond interaction is corroborated by a previous observation in which naphthalene sulfonamide molecules did not display selective inhibition against ePKs until the all-carbon naphthalene ring is substituted with an isoquinoline [27].

In contrast to APH(3′)-IIIa in which the aminoethyl tail adopts an extended conformation, this groups adopts the same conformation and is placed in the equivalent area as that in APH(9)-Ia. The aminoethyl tail found in the CK1 structure bends back toward the sulfonyl group and forms an intramolecular interaction between the terminal nitrogen atom and the equatorial sulfonyl oxygen atom. Deviating slightly from the binding mode of CKI-7 to APH(9)-Ia, the contact between the N_β_ of the aminoethyl and carbonyl of Leu88 located in the linker of the enzyme is achieved via a water molecule, compared to a direct interaction observed in APH(9)-Ia.

### Implications for Inhibitor Design and Adjuvant Development

Previous studies, together with the present report, indicate that CKI-7 can be exploited as a lead compound for APH inhibitor development. Two requirements must be met, however, to make such an inhibitor potentially useful in drug-adjuvant therapy: (1) despite the structural homology between APHs and ePKs, an inhibitor should be selective for the bacterial resistance enzymes. (2) Given that resistant pathogens can harbor multiple APH enzymes, it is desirable that such an inhibitor has a broad-spectrum of activity against various APHs. It is encouraging to note that CKI-7 is only able to effectively inhibit a few ePKs [28] while it can modestly inhibit at least three different APHs. This suggests that the two requirements for an adjuvant application might be achievable. The structural data for the three kinase-CKI-7 complexes provides further information to assess feasibility.

With respect to achieving selectivity for APHs *vs.* ePKs, the three kinase-CKI-7 structures reveal ample differences in binding, as also reflected in the variance in the enzymes' K_i_ values, to confidently suggest that inhibitors based on the isoquinoline scaffold can be developed for either APH(3′)-IIIa or APH(9)-Ia that do not bind to CK1 and likely other ePKs. This confidence is fueled by the extensive expertise in the development of specific inhibitors to the ATP-binding pocket of ePKs, which has shown that sequence divergence in this part of the enzyme is a good predictor of whether selectivity can be obtained [24], [29], [30], [31] The ATP-binding pocket of APH(3′)-IIIa is distinct from CK1 and other ePKs as it a possesses a large aromatic residue (Tyr42) that forms stacking interactions with the isoquinoline moiety. Additionally, the combination of a large nonpolar gatekeeper residue (Met90), a nonpolar residue in the entrance pocket (Leu97) and the absence of a glycine residue in the hinge region that can induce backbone conformation changes, results in a pocket that is predicted to be suitable for selective targeting. Similarly, the nucleotide-binding pocket in APH(9)-Ia shares many of these distinguishing features with APH(3′)-IIIa, though it has a polar residue in the entrance pocket (Asn107), making this pocket somewhat less different from a few ePKs.

Regarding the feasibility of obtaining an inhibitor with broad-specificity against various APHs, the structural data presented here is less encouraging. The differences observed in the nucleotide-binding pocket between APH(3′)-IIIa and APH(9)-Ia are similar in magnitude as those between APH(3′)-IIIa and CK1. This in itself is not necessarily problematic as long as those differences do not compromise their distinctive features from ePKs. However, one of the most distinguishing features of many APHs, i.e. the aromatic residue that forms stacking interactions with the isoquinoline moiety, has differential impacts on inhibitor binding in the two APH structures studied. As a result, the orientation and conformation of CKI-7 observed in APH(9)-Ia mirrors what is seen in CK1. These observations illustrate the diversity in the architectures of the ATP-binding pocket present within the APH family of enzymes, which will undoubtedly complicate development of broad-spectrum inhibitors. On the other hand, it must be realized that the selection of APH(3′)-IIIa and APH(9)-Ia for these studies impacts the conclusions with respect to the feasibility of adjuvant development. As mentioned, APH(9)-Ia is an atypical member of the APH family, and while it provides valuable insights into the extent of the diversity present in nucleotide-binding pockets of APH enzymes, it does not necessarily reflect the diversity of nucleotide-binding pockets likely encountered in clinical settings. Nonetheless, our observations lead us to contend that a universal inhibitor for all APHs targeting the nucleotide-binding pocket may not be feasible, but the contrasting details between APH(3′)-IIIa, APH(9)-Ia and ePKs suggests that selective inhibitors that target a subset of APHs is attainable.

## Materials and Methods

### Enzyme Inhibition Kinetics

The inhibition constant of CKI-7 was determined by first obtaining the Michaelis-Menten parameter, K_m_^apparent^, at various concentrations of the inhibitor. Steady-state kinetics was examined using the procedure previously reported by McKay et al. [16]. In brief, activity of APH(9)-Ia is monitored by coupling the release of ADP from the phosphorylation step of APH(9)-Ia to the reactions of pyruvate kinase and lactate dehydrogenase. The rate of APH(9)-Ia phosphorylation corresponds to the decrease in absorbance at 340 nm due to the exhaustion of NADH in the conversion from pyurvate to lactate by lactate dehydrogenase. The enzyme assay was performed in the presence of five concentrations of CKI-7 (0, 50, 100, 400, and 800 µM) at near saturation level of the substrate spectinomycin (1 mM) and a range of ATP concentrations. All assays were conducted in triplicates with a final reaction volume of 200 µL and measured using a SPECTRAMax 190 (Molecular Devices) 96-well plate spectrophotometer. K_m_^apparent^ was calculated from equation 1 with non-linear regression using Grafit version 5.0.1 software.
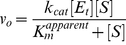
(1)


The inhibition constant, K_I_, was determined from a plot of K_m_^apparent^ versus CKI-7 concentrations, where the x-intercept gives the value of –K_I_.
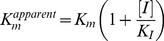
(2)


### Crystallization and Data Collection

APH(3′)-IIIa was expressed and purified using previously established methods [16]. Procedures for the crystallization and data collection of APH(3′)-IIIa-CKI-7 complex have been published [32]. Briefly, crystals of APH(3′)-IIIa-CKI-7 complex were grown using polyethylene glycol (PEG) 3000 as the precipitant and diffracting crystals were obtained by successive cycles of microseeding. Data were collected under cryogenic conditions from a single crystal at beamline X8C of the National Synchrotron Light Source, Brookhaven National Laboratory (Upton, NY), equipped with an ADSC Quantum CCD detector.

APH(9)-Ia was expressed and purified using protocols reported [33]. Details of the initial crystallization strategy of APH(9)-Ia in the presence of CKI-7 has been described [33]. Crystals were obtained in drops containing 2 µL of APH(9)-Ia-CKI-7 mixture, 2 µL of well solution consisting 15% PEG 3350 and 0.2 M calcium acetate, and 0.4 µL of 50 mM manganese chloride. Diffraction data from a single crystal were collected under cryogenic conditions on a Rigaku rotating copper anode generator equipped with Osmic confocal optics and an R-AXIS IV^++^ image-plate detector system. Prior to data collection, the crystal was soaked briefly in a cryoprotectant solution containing 20% PEG 3350, 0.2 M calcium acetate and 1 M sodium formate. Processing of diffraction data for both crystals was performed using HKL2000 [34]. A summary of data collection and processing statistics is shown in Table 1.

**Table 1:** Data collection and refinement statistics.

Protein	APH(3′)-IIIa	APH(9)-Ia
Space group	P2_1_2_1_2_1_	P3_1_21
Cell dimensions		
a (Å)	49.84	74.30
b (Å)	91.85	74.30
c (Å)	131.2	137.0
Resolution (Å) a	2.15 (2.23-2.15)	2.90 (3.00-2.90)
Reflections observed	135045	73147
Unique reflections	32199	10144
Redundancy a	4.2 (1.9)	7.2 (7.5)
Completeness (%) a	95.8 (75.1)	99.0 (99.8)
R_sym_ b (%) a	0.070 (0.240)	0.072 (0.441)
Mean I/σ(I) a	14.3 (3.4)	18.8 (4.2)
R_cryst_ c/R_free_ d	0.189/0.235	0.221/0.279
Number of atoms		
Protein	4340	2657
Inhibitor	36	18
Ions	2 Ca^2+^	1 Ni^2+^
Water	281	25
rmsd		
Bond length (Å)	0.008	0.008
Bond angles (°)	1.1	1.4
Average B factors (Å^2^)		
Protein	32.8	58.1
Inhibitor	36.7	55.2
Ions	43.7	53.7
Water	33.5	34.5

a Values in parentheses refer to reflections in the highest resolution shell.

b R_sym_  =  Σ_hkl_ Σ_i_ |I_i_(hkl)- <I(hkl)>|/Σ_hkl_ Σ_i_ I_i_(hkl), where <I(hkl)> is the average intensity of equivalent reflections and the sum is extended over all measured observations for all unique reflections.

c R_cryst_  =  Σ_hkl_ (|F_obs_| - |F_calc_|)/Σ_hkl_ |F_obs_|, where |F_obs_| is the observed and |F_calc_| is the calculated structure factor amplitude of a reflection.

d R_free_ was calculated by randomly omitting 10% of the observed reflections from the refinement.

### Structure Determination and Refinement

Despite different crystal growth conditions, APH(3′)-IIIa-CKI-7 crystal was isomorphous with APH(3′)-IIIa-ADP crystals. Hence, the APH(3′)-IIIa-ADP structure (PDB ID 1J7L) [8], excluding the ligand and water molecules, was used as the starting model for the refinement of APH(3′)-IIIa-CKI-7 complex in the Crystallography and NMR System (CNS) program [35]. After rigid body refinement and an initial cycle of positional and grouped thermal factor refinement, one molecule of CKI-7 was modeled in each active site in the space where σ_a_-weighted difference maps (2F_o_-F_c_ and F_o_-F_c_) displayed positive electron density. The stereochemical parameters for CKI-7 used in subsequent refinement were based on the conformation of the inhibitor found in the crystal structure of casein kinase 1 (PDB ID 2CSN) [25] in conjunction with values from the energy minimized conformation obtained from the molecular mechanics program MM2 [36], [37] implemented in CambridgeSoft Chem3D software. Upon inspection, several regions required remodeling due to considerable deviations from the difference density maps. Cycles of positional and individual thermal factor refinement was alternated with manual refitting using the program O [38] and incorporation of solvent molecules. The process was continued in the programs PHENIX [39] and COOT [40] until no further improvement in model statistics could be obtained.

The structure of CKI-7-bound APH(9)-Ia was solved by molecular replacement using Phaser [41] as implemented in the CCP4 software suite [42] and the apo structure of APH(9)-Ia (PDB ID 3I1A) as the starting model. A solution of the proper position and orientation of the inhibitor-bound molecule was obtained only when the search model was input as two entities – the N-terminal lobe (residues 3–199) and the C-terminal lobe (residues 200–330). Following several cycles of positional and grouped B-factor refinement, a molecule of CKI-7 was placed in the nucleotide-binding site according to the observed positive difference maps. The same stereochemical parameters used for APH(3′)-IIIa-bound CKI-7 were applied to the APH(9)-Ia-bound inhibitor for structure refinement. Iterative cycles of positional and individual thermal factor refinement using CNS are intercalated with manual adjustments along with the addition of water molecules. Several regions (residues 27–40, 53–60, 89–97, and 297–309) that display substantial difference between the model and electron density were manually rebuilt using the program O [38] according to difference density maps and simulated annealing omit maps. Final refinement statistics for the inhibitor-bound structures of both APH enzymes are summarized in Table 1.
